# Interaction of Temporin-L Analogues with the *E. coli* FtsZ Protein

**DOI:** 10.3390/antibiotics10060704

**Published:** 2021-06-11

**Authors:** Angela Di Somma, Carolina Canè, Antonio Moretta, Angela Duilio

**Affiliations:** 1 Department of Chemical Sciences, Università Federico II di Napoli, 80126 Napoli, Italy; angela.disomma@unina.it (A.D.S.); carolina.cane@unina.it (C.C.); 2 Departiment of Science, Università degli Studi della Basilicata, 85100 Potenza, Italy; antonio.moretta@unibas.it

**Keywords:** antimicrobial peptide, Temporin-L, FtsZ inhibition, Temporin analogues

## Abstract

The research of new therapeutic agents to fight bacterial infections has recently focused on the investigation of antimicrobial peptides (AMPs), the most common weapon that all organisms produce to prevent invasion by external pathogens. Among AMPs, the amphibian Temporins constitute a well-known family with high antibacterial properties against Gram-positive and Gram-negative bacteria. In particular, Temporin-L was shown to affect bacterial cell division by inhibiting FtsZ, a tubulin-like protein involved in the crucial step of Z-ring formation at the beginning of the division process. As FtsZ represents a leading target for new antibacterial compounds, in this paper we investigated in detail the interaction of Temporin L with *Escherichia coli* FtsZ and designed two TL analogues in an attempt to increase peptide-protein interactions and to better understand the structural determinants leading to FtsZ inhibition. The results demonstrated that the TL analogues improved their binding to FtsZ, originating stable protein-peptide complexes. Functional studies showed that both peptides were endowed with a high capability of inhibiting both the enzymatic and polymerization activities of the protein. Moreover, the TL analogues were able to inhibit bacterial growth at low micromolar concentrations. These observations may open up the way to the development of novel peptide or peptidomimetic drugs tailored to bind FtsZ, hampering a crucial process of bacterial life that might be proposed for future pharmaceutical applications.

## 1. Introduction

The emergence of resistant bacteria is rising to dangerously high levels worldwide. New resistance mechanisms are emerging and spreading globally, threatening our ability to treat common infectious diseases. Several well-known infections are becoming harder, and sometimes impossible, to treat, as the efficacy of antibiotics is hampered by antimicrobial resistance. The onset of antibiotic resistance is accelerated by the misuse and overuse of antibiotics as these drugs are often overprescribed by health workers and overused by the public [1].

The research and development of new therapeutic agents to fight bacterial infections should then be prioritized. In this respect, growing interest has recently focused on the investigation of antimicrobial peptides (AMPs) as new possible human therapeutics, alone or in combination with current antibiotics. AMPs are produced by several tissues and cell types in a variety of plants and in animal species like insects, amphibians, and vertebrates [2,3]. AMPs are currently considered promising candidates as alternative therapeutic agents because of their broad spectrum of activity against several different microorganisms, their ability to inhibit bacterial growth and decrease the development of bacterial resistance [4,5].

Among AMPs of natural origin, the amphibian Temporins represent one of the largest families with high antibacterial properties against a number of Gram-positive and Gram-negative bacteria that cause infections such as skin diseases, meningitis and urinary tract infections in human beings [6]. Recently, the mechanism of action of Temporin-L in *E. coli* was completely elucidated, identifying the FtsZ protein as its specific intracellular target [7]. FtsZ belongs to the divisome complex and plays an important role in orchestrating bacterial cell division [8]. Assembly and activation of the divisome machinery are precisely coordinated by the GTPase FtsZ, which polymerizes into a dynamic ring defining the division site, recruiting downstream proteins, and directing peptidoglycan synthesis to drive constriction [9]. Temporin-L is able to specifically bind the FtsZ protein, inhibiting its GTPase activity with a competitive mechanism then hampering cell division.

Since FtsZ is responsible for a crucial biological event of bacterial life and it is absent in humans, this protein might represent a good target for the rational design of new antimicrobial molecules. In this paper, we investigated the interaction of two new Temporin-L analogues with *E. coli* FtsZ. The interaction of native Temporin-L and the two mutated peptides with the protein was evaluated both in silico, through molecular docking analysis, and in vitro by enzymatic and polymerization assays, and the binding parameters were measured by fluorescence analyses. Finally, the antibacterial activity of the two mutated peptides was evaluated by determination of the minimum inhibitory concentration (MIC) compared to native Temporin-L.

Investigation of FtsZ inhibition by antimicrobial peptides might open up the way to the development of new peptide or peptidomimetic drugs able to impair a crucial process of bacterial life by targeting a specific molecule in a reversible way thus avoiding (or slowing down) the onset of antidrug resistance.

## 2. Results

### 2.1. Design of the Mutated Temporin-L Peptides and Molecular Docking Analysis

The antimicrobial peptide Temporin-L (TL) was recently demonstrated to act as a competitive inhibitor toward the *E. coli* FtsZ protein, thus impairing cell division [7]. As FtsZ might represent a good target for the development of new drugs, we were stimulated to investigate this peptide-protein interaction in more detail by docking simulations. FtsZ and TL were modelled using the I-TASSER web server originating the structural models shown in Supplementary Figure S1a,b, respectively. The corresponding model parameters, C-score, TM-score, and RMSD, are listed in Table 1.

Molecular docking experiments were performed to evaluate the FtsZ-TL complexes using the previously obtained models and the PatchDock server; the putative structures were then refined by the FireDock server. Through the PDBsum server, the main interactions at the protein-peptide interface and the involved amino acids could be identified.

**Figure 1:**
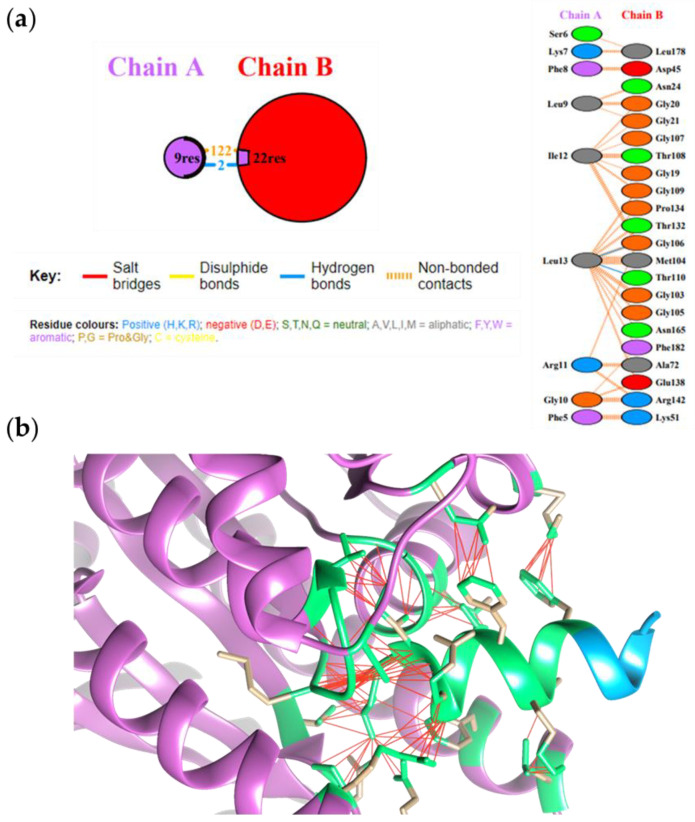
(**a**) Interactions identified at the FtsZ (Chain B)-TL (Chain A) interface. (**b**) Molecular docking of FtsZ-native TL complex. FtsZ is shown in light magenta, native TL peptide in light blue. The amino acids involved at the peptide-protein interface are in green. Interactions are reported with red lines.

Figure 1a,b shows the occurrence of 112 noncovalent interactions and 2 hydrogen bonds involving TL Leu13-FtsZ Gly106 and TL Leu13-FtsZ Thr110 and the putative structure of the complex highlighting the interactions involved at the interface.

The stability of the complex was evaluated by calculating the global energy, the attractive and repulsive van der Waals forces and the atomic contact energy (ACE). The corresponding values are listed in Table 2 together with the predicted ΔG suggesting the formation of a stable peptide-protein complex.

The investigation of the FtsZ-TL complex model suggested by docking simulations prompted us to design specific TL analogues with the aim of improving the peptide−protein interactions and increasing the ability of the peptide to inhibit FtsZ activity. Two mutated Temporin-L peptides were then designed, tailoring the amino acid mutations on the basis of a detailed examination of the docking model and the antibacterial prediction scores calculated by the CAMP (Collection of Antimicrobial Peptides) database. The amino acid sequences of the two mutated TL analogues, TRIL and TRILF are listed in Table 3 together with the prediction scores of their antimicrobial properties generated by using the SVM, DA, RF, and ANN algorithms available in the CAMP database. The data have been compared with those obtained for the native TL.

A similar or higher putative antibacterial activity for the two TL analogues compared to native TL was predicted by all algorithms.

**Figure 2:**
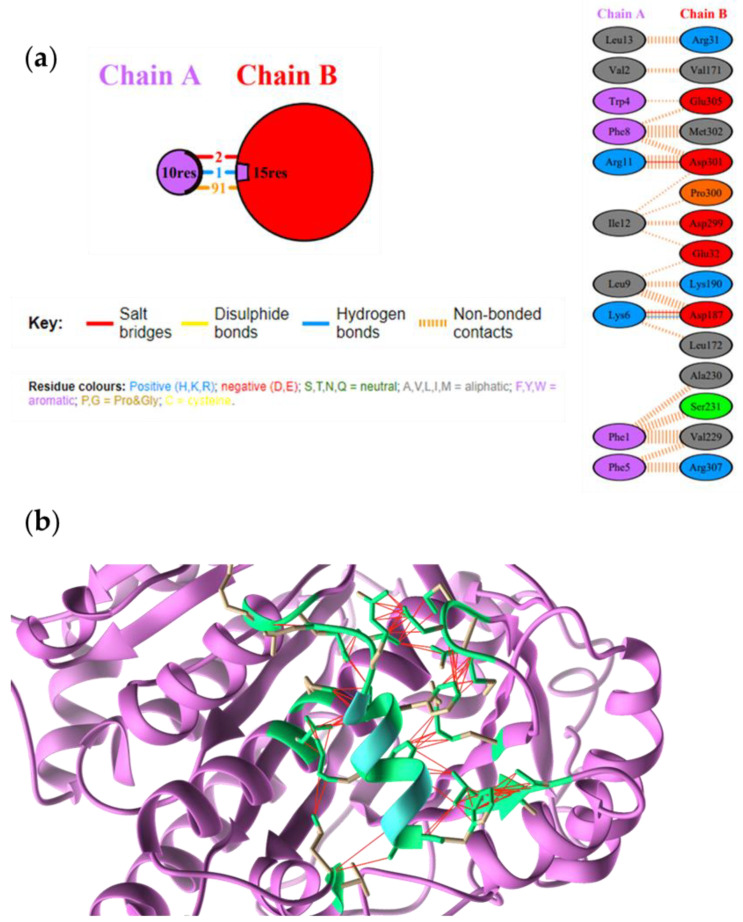
(**a**) Interactions identified at the FtsZ (Chain B)-TRIL (Chain A) interface. (**b**) Molecular docking of FtsZ−native TRIL complex. FtsZ is shown in light magenta, native TL peptide in light blue. The amino acids involved at the peptide-protein interface are in green. Interactions are reported with red lines.

**Figure 3:**
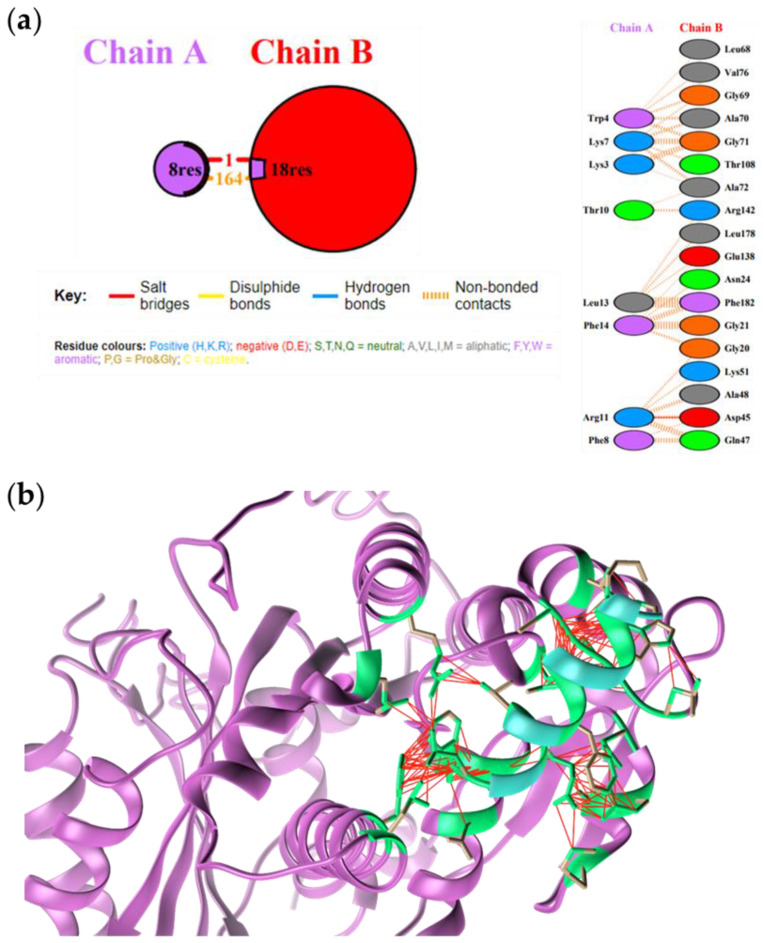
(**a**) Interactions identified at the FtsZ (Chain B)-TRILF (Chain A) interface. (**b**) Molecular docking of FtsZ−native TRILF complex. FtsZ is shown in light magenta, native TL peptide in light blue. The amino acids involved at the peptide-protein interface are in green. Interactions are reported with red lines.

The models of the two new TL analogues were then built up (Supplementary Figure S1c,d respectively) and their modeling parameters are listed in Table 1. Molecular docking simulations were performed using the same conditions described above and the main interactions at the protein-peptide interface and the involved amino acids identified in both the FtsZ-TRIL and FtsZ-TRILF complexes are shown in Figure 2 and Figure 3, respectively.

A total of 91 noncovalent interactions were detected at the protein-peptide interface for the FtsZ-TRIL complex together with two salt bridges involving TRIL Lys6-FtsZ Asp187 and TRIL Arg11-FtsZ Asp30, and a hydrogen bond between TRIL Lys6 and FtsZ Asp187 (Figure 2).

The FtsZ-TRILF complex model showed 164 noncovalent interactions and the hydrogen bond between TRILF Arg11 and FtsZ Asp45 occurring at the protein-peptide interface (Figure 3).

The stability parameters of both complexes were calculated and are listed in Table 2, together with the predicted ΔG values. The in silico analyses indicated that both TL analogues were able to form stable peptide-protein complexes with the FtsZ protein suggesting that they were good candidates as inhibitors of its GTPase activity.

### 2.2. Binding of the Temporin-L Analogues to FtsZ

**Figure 4:**
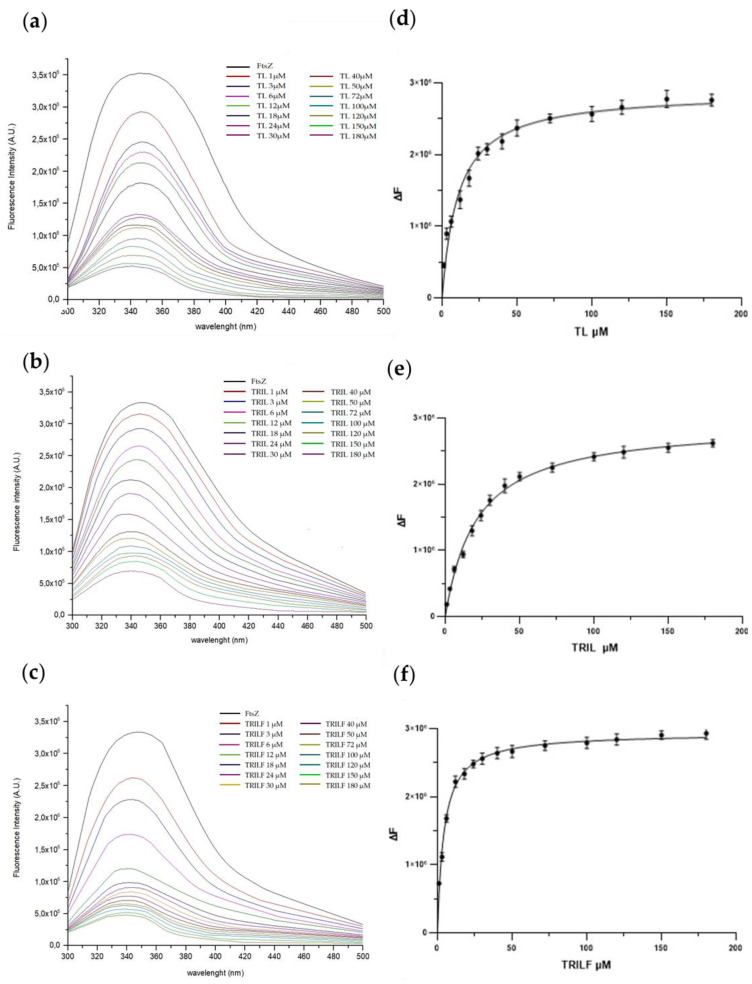
Left panel: Spectrofluorimetric titration of FtsZ (12 μM) in complex with native TL (**a**), TRIL (**b**) and TRILF (**c**) from 1 to 180 μM, was performed by monitoring the emissions at 347 nm at 20 °C. Right panel (**d**–**f**): Diagram plots extrapolated from fluorescence data. The experiments were performed in duplicate and the standard deviation is reported as error bars.

As docking calculations suggested the formation of putative FtsZ-TRIL and FtsZ-TRILF complexes, the binding of the two peptides to the FtsZ protein was investigated by fluorescence experiments. A recombinant form of FtsZ was produced in *E. coli*, purified and used in binding and enzymatic assays [7]. A significant decrease of fluorescence intensity of FtsZ with the increased concentrations of antimicrobial peptides was observed in both assays, as shown in Figure 4 where the data from the FtsZ−native TL complex are also reported for comparison.

Moreover, a progressive, although limited, shift from 347 to 340 nm of the maximum emission wavelength could also be detected, demonstrating that the antimicrobial peptides could interact with FtsZ and alter its intrinsic fluorescence.

The dissociation constant of the three complexes were calculated from the fluorescence experiments. The Kd values for the FtsZ-TL and FtsZ-TRIL complexes were determined as 11.0 ± 1.0 µM and 21.4 ± 1.1 µM, respectively, confirming a good interaction between the peptides and the FtsZ protein. Moreover, an even better result was obtained for the FtsZ-TRILF complex whose dissociation constant was measured as 4.3 ± 0.2 µM, indicating that the insertion of a Phe residue at the C-terminus of TL increased the stability of the interaction with the target protein.

### 2.3. Effect of TL Analogues on the GTPase Activity of FtsZ

**Figure 5:**
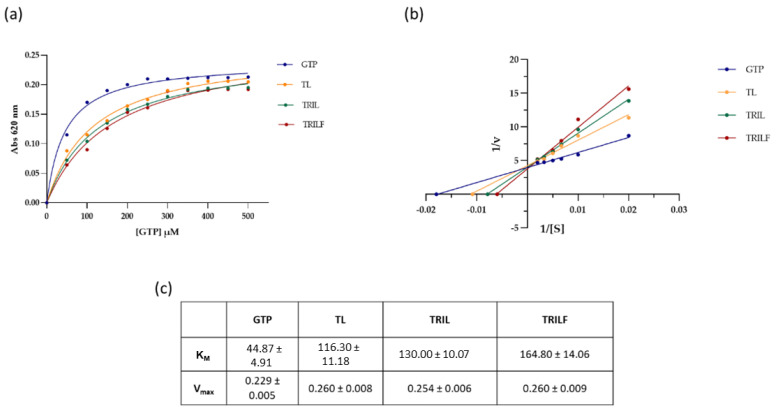
(**a**) Enzymatic activity of recombinant FtsZ (12 µM) was performed in 25 mM PIPES/NaOH (pH 6.8), 20 mM MgCl_2_, in the absence (blue line) and in the presence of 35 µM TL (yellow line), TRIL (green line) and TRILF (red line) using GTP as substrate. The reaction was performed for 10 min and the Pi release was determined by measuring the absorbance at 620 nm following 25 min incubation. (**b**) Lineweaver−Burk plots from which the K_M_ constants were calculated. (**c**) Calculated K_M_ constants and V_max_ for the enzymatic assays. The experiment was performed in duplicate and presented as mean ± standard error.

The putative effect of the two TL analogues on FtsZ GTPase activity was evaluated by enzymatic assays. The purified recombinant protein was incubated with GTP in the presence of either TRIL or TRILF peptides (35 µM) and the GTPase activity was monitored in comparison with the untreated protein at different GTP concentrations. The results showed that the FtsZ activity was inhibited by both peptides in a dose-dependent manner, confirming a functional interaction of the antimicrobial peptides with the GTPase. Figure 5 shows the kinetic profiles of the enzymatic assays in the absence and in the presence of the peptides.

Kinetic parameters were calculated from the Lineweaver-Burk plot (Figure 5b) showing an increase of the apparent K_M_ for both peptides, 130.0 ± 10.1 μM for TRIL and 164.8± 14.1 μM for TRILF as compared to 44.9 ± 4.9 μM in the absence of the peptides (Figure 5c). These data were compared to the kinetic parameters measured for native TL, 116.3 ± 11.2 μM, indicating that both TRIL and TRILF exerted a stronger inhibitory effect than TL decreasing the affinity of the enzyme for its natural substrate GTP by 65% and 73% respectively. It should be underlined that in all essays, the V_max_ value remained unchanged, demonstrating that both TL analogues adopted a competitive inhibitory mechanism. The enzymatic activity of FtsZ was also tested in the presence of a different peptide Magainin-2 (Mag-2). Under these conditions, it was clearly demonstrated that this peptide had no effect on the GTP activity since the kinetic parameters were unaltered (Supplementary Figure S2).

### 2.4. Effect of TL Analogues on the Polymerization of FtsZ

Since FtsZ is known to polymerize into long filaments in the presence of GTP in vitro, we were prompted to investigate the effect of the TL analogues on the polymerization of FtsZ. A simple polymerization assay was then performed by incubating FtsZ with GTP (1 mM) in the absence and in the presence of increasing concentrations of both native TL and the two TL analogues. The FtsZ filaments were purified by centrifugation and analyzed by SDS-PAGE and the amount of protein pelleted was determined by densitometric analysis of the corresponding Coomassie stained gel band.

**Figure 6:**
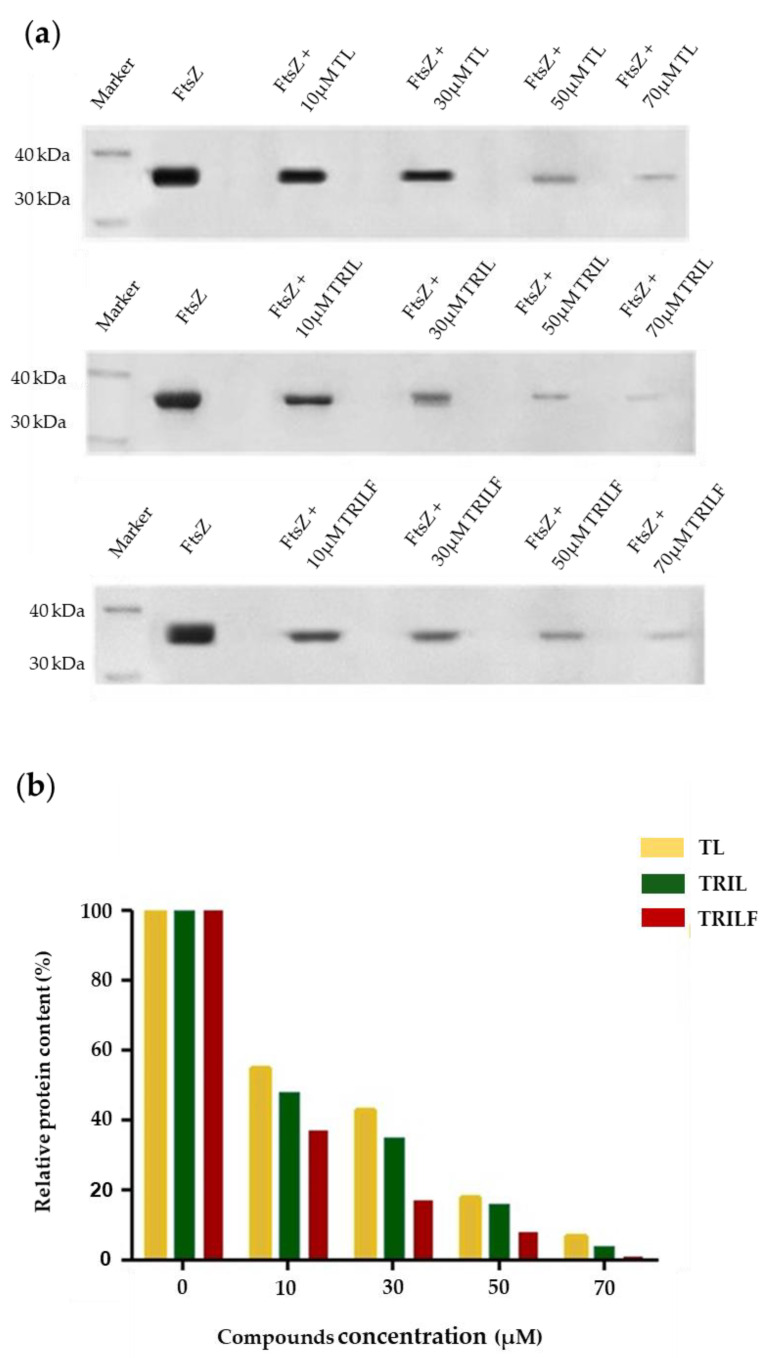
Polymerization assay of recombinant FtsZ (12 µM) was carried out in 25 mM PIPES/NaOH, pH 6.8 and 1 mM of GTP in the absence and in the presence of different concentrations of TL, TRIL and TRILF. (**a**) SDS-PAGE (12.5%) of protein pellet after polymerization reaction. (**b**) Densitometric analysis of SDS-PAGE in (**a**), showing the percentage of FtsZ polymerization in the absence and in the presence of TL and TL analogues.

Figure 6 clearly shows that the amount of polymerized FtsZ decreased when increasing concentrations of either native TL or the two TL analogues were added in the assay. Moreover, FtsZ polymerization was impaired in a dose-dependent manner with TRILF showing the highest inhibition ability, reaching 82% inhibition at 30 µΜ, while TL and TRIL showed 42% and 35% inhibition under the same conditions (Figure 6b).

### 2.5. Antimicrobial Activity of TRIL and TRILF

Finally, we evaluated the in vivo antimicrobial activity of TRIL and TRILF on *E. coli* cells. First the minimal inhibitory concentrations (MIC) of both TL analogues were determined by the lowest concentration showing no visible growth after 24 h of incubation, demonstrating that both peptides were highly active against *E. coli* at low micromolar concentrations (MIC = 8 μM).

**Figure 7:**
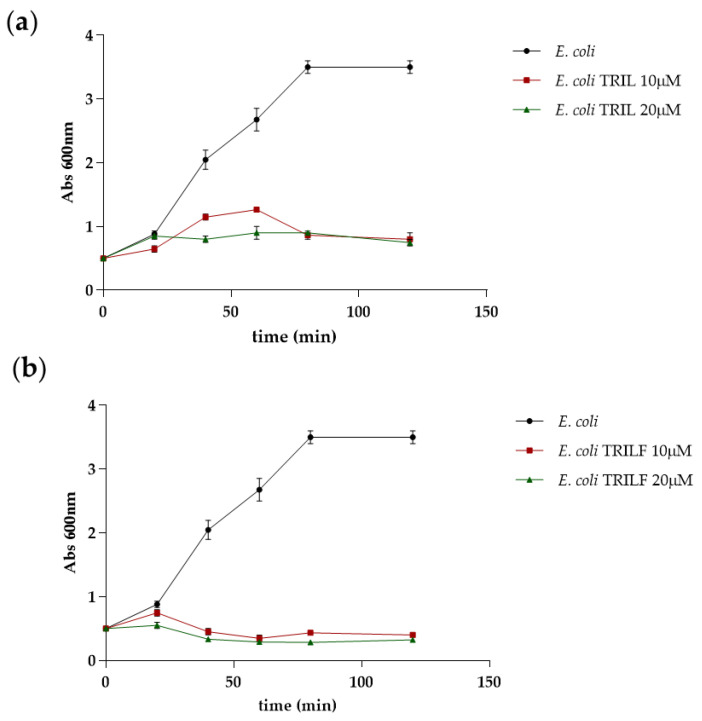
Growth profiles of *E. coli* cells in the presence of different concentrations of TRIL (**a**) and TRILF (**b**) analogues were obtained by monitoring cells for 120 min at 600 nm. The growth profile in the absence of the peptides is shown for comparison. Experiments were run in duplicate and the standard deviation is reported as error bars.

Next, the growth profile of the *E. coli* cells was evaluated in the presence of different concentrations of the two peptides, as shown in Figure 7.

The growth profiles demonstrated that both peptides are able to inhibit bacterial cell growth with TRILF showing a greater effect being able to totally impair cell growth at 10 µM.

In addition, the effect of TRIL and TRILF on *E. coli* was also investigated using a conditional Δftsz mutant strain in which the gene coding for FtsZ was silenced when the cells were grown at 42 °C.

**Figure 8:**
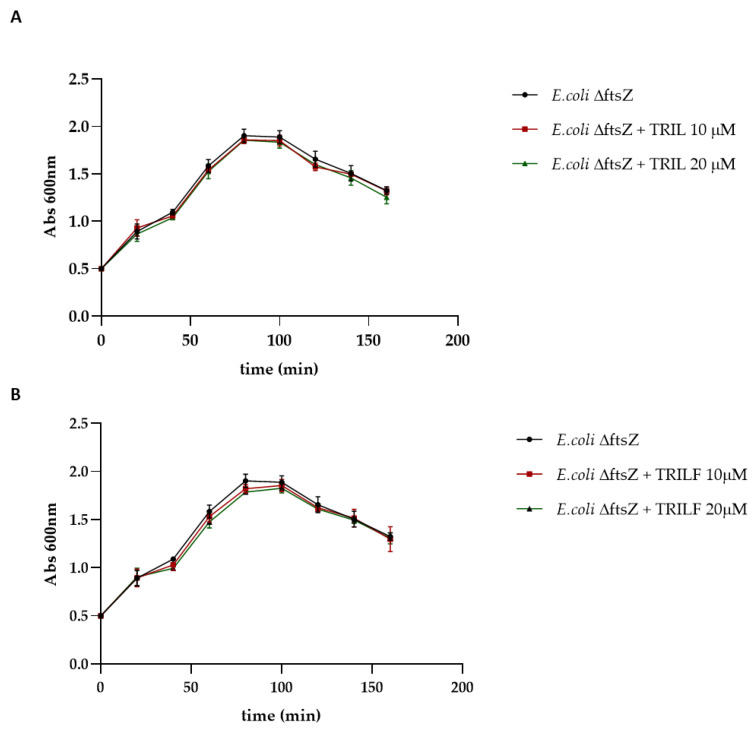
Growth profiles of ΔftsZ strain in the absence and in the presence of different concentrations of TRIL (**A**) and TRILF (**B**) analogues obtained by monitoring cells for 300 min at 600 nm. Experiments were run in duplicate and the standard deviation is reported as error bars.

The growth profile of the ΔftsZ strain was evaluated at 42 °C in the absence and in the presence of different concentrations of the two peptides (Figure 8). The growth profiles of the untreated and the treated ΔftsZ mutant strain were almost superimposable, indicating that the two peptides did not exert any effect on the mutant strain. These results suggested that the two TL analogues could affect cell growth only in the presence of FtsZ, confirming this enzyme as their specific target.

## 3. Discussion

Temporin L belongs to a well-known family of small, linear antibiotic peptides with intriguing biological properties and displaying the highest activity among the temporins studied so far, against both bacterial and fungal strains. Since the discovery of temporins in 1996 [10], these peptides have been considered as potential pharmaceutic candidates [11], although their mechanism of action is mostly unknown. In particular, the interaction of Temporin L with both model biomembranes [12], liposomes of different lipid compositions [13] and bacterial membranes [14,15] was carefully examined.

Recently, we investigated the mechanism of action of Temporin L on *E. coli*, showing that the peptide enters the cells interacting with a specific intracellular target, FtsZ, a tubulin-like protein endowed with GTPase activity. FtsZ is a key factor in the divisome complex and inhibition of its GTPase activity by TL impairs cell division [7]. Nowadays, Ftsz is recognized as a leading target in the search for new antibacterial compounds, since it is an essential protein for cell division in most bacteria and is absent in humans [16]. A large number of FtsZ inhibitors, including peptides, natural products, and other synthetic small molecules, have been proposed which might lead to the discovery of novel FtsZ-targeting clinical drugs [17].

On this basis, we were stimulated to examine in more detail the interaction of Temporin L with *E. coli* FtsZ. A model of the peptide-protein complex was then constructed by docking simulation, showing a good thermodynamic stability, and the main interactions occurring at the complex interface were predicted. Docking calculations suggested that TL interacts with FtsZ close to the GTP binding pocket, making interactions mainly with helices H1, H6, H7 and H8 and strands S4 and S5.

Using the TL-FtsZ model as a guideline, two TL analogues were designed in an attempt to increase peptide-protein interactions and to better understand the structural determinants leading to FtsZ inhibition. Several Temporin L analogues have already been proposed and their properties tested with the aim of increasing the antimicrobial activity of the peptide and decreasing its cytotoxic effects [18]. We focused on TL analogues endowed with higher capability of binding to FtsZ and inhibiting its enzymatic activity.

As suggested by the docking model, all the hydrophobic amino acids have not been replaced because they are involved in a patchwork of hydrophobic interactions with the FtsZ protein. Furthermore, the positively charged residues (Arg and Lys) were left unchanged since they are essential for antibacterial activity, allowing electrostatic interactions with bacterial membranes. Sequence mutations were then introduced at positions 3, 6 and 10 of TL where Lys, Lys and Thr replaced the naturally occurring Gln, Ser and Gly respectively, to ameliorate peptide-protein contacts within the GTP binding site. A further Phe residue was added at the C-terminus of the second TL analogue as the model displayed a sufficiently large cavity to accommodate the hydrophobic ring of the Phe residue. 

The TRIL and TRILF analogues were modeled in a complex with *E. coli* FtsZ and the docking calculations predicted an increased stability of both complexes, indicating the correctness of the tailored sequence modifications. Model predictions were then confirmed by investigating the interaction of the two TL analogues with the protein target and their ability to inhibit both FtsZ assembly and GTPase activities on experimental bases.

Fluorescence binding assays performed at different concentrations of TRIL and TRILF analogues demonstrated that both peptides can bind FtsZ, originating stable protein-peptide complexes with dissociation constants in the low micromolar range, thus confirming the docking predictions. A functional investigation of the TL analogues was performed by both enzymatic assays and FtsZ polymerization tests. Both peptides were found to be competitive inhibitors of the GTPase activity of FtsZ, showing a 3–4 times increase in the K_M_ of the enzyme for GTP. Moreover, the TL analogues impaired FtsZ polymerization in a dose dependent manner with TRILF showing the highest inhibition capability. Finally, the antibacterial properties of the TL analogues were tested in vivo on *E. coli* cells showing that both peptides were able to inhibit bacterial growth at low micromolar concentrations. In Mangoni et al., 2011 [18] different Temporin-L analogues were designed and the authors evaluated their antibacterial activity, obtaining MIC values ranging from 3 to >48 μM. These results indicate that the MIC values obtained for TRIL and TRILF represent a good result, considering that these sequences also show an excellent interaction with the FtsZ protein target.

Our data suggest that a careful inspection of peptide-FtsZ complexes might be instrumental in understanding the main structural determinants leading to enzyme inhibition. Moreover, the competitive inhibitory mechanism exerted by the TL peptides and the K_D_ values of the corresponding complexes suggest a reversible mode of action that might impair or delay the onset of bacterial resistance. These observations may open up the way to the development of novel peptide or peptidomimetic drugs tailored to bind FtsZ, exerting a competitive inhibitory activity on this crucial enzyme that might be proposed for future pharmaceutical applications.

## 4. Materials and Methods

### 4.1. Design of the Temporin-L Analogues and Molecular Docking Analyses

The FtsZ protein, native TL and the TL analogues were modelled through the I-TASSER webserver [19,20], which associates to each model a C-score whose value ranges from −5 to +2. The higher the value, the better the model. The TM-score and RMSD are known standards for measuring structural similarity between two structures. A TM-score value >0.5 indicates a model of correct topology [21].

The obtained models in .pdb format were exploited to perform the molecular docking analyses using the PatchDock Server [22]. The protein-peptide complexes were then refined with FireDock Server [23], which also gave the global energy, the attractive and repulsive van der Waals (VdW) forces and the atomic contact energy (ACE) values. The PDBsum Server [24,25] was used in order to identify all the interactions and the amino acids involved at the protein-peptide interface. The Gibbs free energy, ΔG, and the dissociation constant, K_d_, of the complex have been predicted using the PRODIGY webserver [26]. All the figures have been generated through UCSF CHIMERA software [27].

The two TL analogues were designed on the basis of the FtsZ-native TL complex predicted by docking analysis trying to increase the peptide-protein interactions. Amino acid substitutions were introduced at positions 3 (Q3K), 6 (S6K) and 10 (G10T), leaving all the hydrophobic residues unchanged. Moreover, a Phe residue was added at the C-terminus of the second TL analogous to increase the hydrophobic interaction occurring at the protein-peptide interface as docking simulation showed the occurrence of a large cavity in that area in the FtsZ-TL complex.

The antibacterial activity predictions scores of native TL were calculated by the CAMP (Collection of Antimicrobial Peptides) database [28], which exploits four different machine-learning algorithms: Support Vector Machine (SVM), Discriminant Analysis (DA), Artificial Neural Network (ANN) and Random Forest (RF). The results are given in the form of a numerical score except for the Artificial Neural Network (ANN), where the result is indicated as AMP (antimicrobial) or NAMP (non-antimicrobial).

### 4.2. Recombinant Production of FtsZ

Recombinant *E. coli* FtsZ was overexpressed and purified from *E. coli* BL21 strain as described previously [7]. The purity of the protein was analyzed by SDS-PAGE 12.5% and its primary structure was validated by MALDI mapping strategy on a 5800 MALDI-TOF/TOF instrument (ABI Sciex, Foster City, CA, USA). The concentration of purified FtsZ was determined by Bradford’s method [29], using BSA as a standard. The protein was stored at −80 °C.

### 4.3. Binding Experiment

Fluorescence experiments were performed using a Fluoromax-4 spectrofluorometer from Horiba Scientific, using 1 cm optical path-length quartz cell under controlled temperature conditions (Peltier control system at 20 °C). The sample was analyzed, monitoring the fluorescence intensity of aromatic residues with an excitation wavelength of 280 nm. The wavelength range explored was 295–500 nm. FtsZ protein at a concentration of 12 μM was excited at 280 nm (slit 4 nm) and the emission was monitored at 347 nm (slit 4 nm) without and in the presence of increasing concentrations of TL, TRIL and TRILF (from 1 to 180 μM) in a high voltage mode. All experiments were repeated in duplicate. The change in the fluorescence intensity of the reaction set was fitted into “one site-specific binding” equation of GraphPad Prism 5 (GraphPad Software, San Diego, CA, USA).

### 4.4. GTPase Activity Assay

The amount of Pi released during the assembly of FtsZ was measured using BIOMOL Green phosphate reagent (Biomol, Milan, Italy) as described earlier [7]. Briefly, FtsZ (6 µM) was incubated with different concentrations of GTP, ranging from 0 µM to 250 µM, either in the absence or in the presence of 35 µM TL, TRIL and TRILF in 25 mM PIPES/NaOH, pH 6.8 for 30 min at 30 °C. The reaction was performed for 10 min and stopped by addition of 100 µL BIOMOL Green reagent. The Pi release was determined after incubation at 25 °C for 25 min by measuring the absorbance at 620 nm. The background was subtracted from all the readings. The experiment was performed in duplicate. Kinetic parameters were fitted by nonlinear regression with GraphPad Prism 4Project.

### 4.5. Polymerization Assay

Polymerization assays were carried out as described in Zheng et al. [30]. *Escherichia coli* FtsZ protein was diluted at a final concentration of 12 µM in 25 mM PIPES/NaOH, pH 6.8. Different concentrations of TL, TRIL and TRILF (10, 30, 50, 70 µM) was added to the protein and the polymerization reaction of FtsZ was carried out in the presence of 1 mM GTP at 25 °C for 1 h. Then the reaction was stopped by centrifugation at 14,000 rpm for 60 min and pellets were re-suspended in 25 mM PIPES/NaOH, pH 6.8 and analyzed by 12.5% SDS-PAGE gel. Gels were stained with Comassie Brilliant Blue and the protein content of bands was measured by densitometric quantification using Quantity One software. Each assay was carried out in triplicates.

### 4.6. Determination of Antibacterial Activity of TRIL and TRILF

The minimum inhibitory concentration (MIC) of TRIL and TRILF was measured by broth microdilution. The cell strain of *E. coli* BL_21_ was incubated overnight in LB at 37 °C. The culture was diluted to obtain a concentration of 0.08 OD_600_/mL in fresh medium and grown at 37 °C for 90 min. At an OD/mL value of 0.5, 50 µL of bacterial suspension was added to ten wells and incubated with serial dilutions of the peptides from an initial concentration of 512 µM. The sterility control well contained 100 μL of LB, while the growth control well contained 100 μL of microbial suspension. The MIC was determined by the lowest concentration showing no visible growth after 24 h of incubation at 37 °C by measuring the Abs at 600 nm. The assay was performed in triplicate.

In addition, *E. coli* cells at 0.5 OD/mL were treated with different concentrations of compounds (1 × MIC and 2 × MIC) and the growth was monitored every 20 min reading optical density at 600 nm. The Δftsz cells (a kind gift from Prof. Miguel’s group) were grown at 37 °C in LB medium supplemented with 50 μg/mL kanamycin and subsequently transferred at 42 °C to silence ftsz and treated with different concentrations of compounds. The ftsz thermonull mutant has been constructed in which the ftsz gene has been deleted from the *Escherichia coli* chromosome while maintaining a wild-type copy of the gene in a thermosensitive plasmid. [31,32,33].

## Figures and Tables

**Table 1:** The obtained C-score, TM-score, and RMSD values for the peptides modelled through the I-TASSER server are listed.

Peptide/Protein	C-Score	TM-Score	RMSD (Å)
FtsZ	−0.55	0.64 ± 0.13	7.9 ± 4.4
TL	0.09	0.73 ± 0.11	0.5 ± 0.5
FV**K**WF**K**KFLTRIL	0.07	0.72 ± 0.11	0.5 ± 0.5
FV**K**WF**K**KFLTRIL**F**	−0.06	0.71 ± 0.12	0.6 ± 0.6

The substituted amino acids are indicated in bold.

**Table 2:** Global energy, attractive and repulsive van der Waals forces, atomic contact energy, and ΔG expressed in Kcal/mol calculated for the FtsZ-TL peptide complexes at 25 °C.

Protein-Peptide Complex	Global Energy (Kcal/mol)	Attractive Van der Waals Forces (KJ/mol)	Repulsive Van der Waals Forces (KJ/mol)
FtsZ−TL	−40.54	−18.63	5.71
FtsZ−TRIL (FV**K**WF**K**KFLTRIL)	−26.84	−30.69	18.88
FtsZ−TRILF (FV**K**WF**K**KFLTRIL**F**)	−30.99	−46.97	24.35

The substituted amino acids are indicated in bold.

**Table 3:** Peptide sequences of the designed TL analogs and the corresponding prediction scores generated by four algorithms (SVM, DA, RF, and ANN). Data from native Temporin L are also listed for comparison.

Peptide	SVM	DA	RF	ANN
Native Temporin L FVQWFSKFLGRIL	0.876	0.899	0.9685	AMP
FV**K**WF**K**KFL**T**RIL	0.997	0.981	0.941	AMP
(TRIL)				
FV**K**WF**K**KFL**T**RIL**F**	0.994	0.973	0.906	AMP
(TRILF)				

The substituted amino acids are indicated in bold.

## Data Availability

Data is contained within the article or Supplementary Material.

## Supplementary Materials

The following are available online at https://www.mdpi.com/article/10.3390/antibiotics10060704/s1, Figure S1: 3D model of FtsZ (a), native Temporin L (b), TRIL analogue (c), and TRILF analogue (d), obtained by ab initio modelling with I-TASSER server. Figure S2: (a) Enzymatic activity of recombinant FtsZ (12 µM) was performed in 25 mM PIPES/NaOH (pH 6.8), 20mM MgCl2, in the absence (blue line) and in the presence of 35µM of Mag-2 peptide (magenta line), using GTP as substrate. The reaction was performed for 10 min and the Pi release was determined by measuring the absorbance at 620 nm following 25 min incubation. (b) Lineweaver Burk plots from which the KM constants were calculated. (c) Calculated KM constants and Vmax for the enzymatic assays. The experiment was performed in duplicate and presented as mean ± standard error.
