# Supplementary material for: Interaction of Temporin-L Analogues with the E. coli FtsZ Protein
Source: Antibiotics (Basel). 2021 Jun 11;10(6):704. doi: 10.3390/antibiotics10060704 (PMC8230800; doi:10.3390/antibiotics10060704)
Supplement: Supplementary file 1 [file antibiotics-10-00704-s001.zip › antibiotics-1226446-supplementary.pdf]

Supplementary Materials

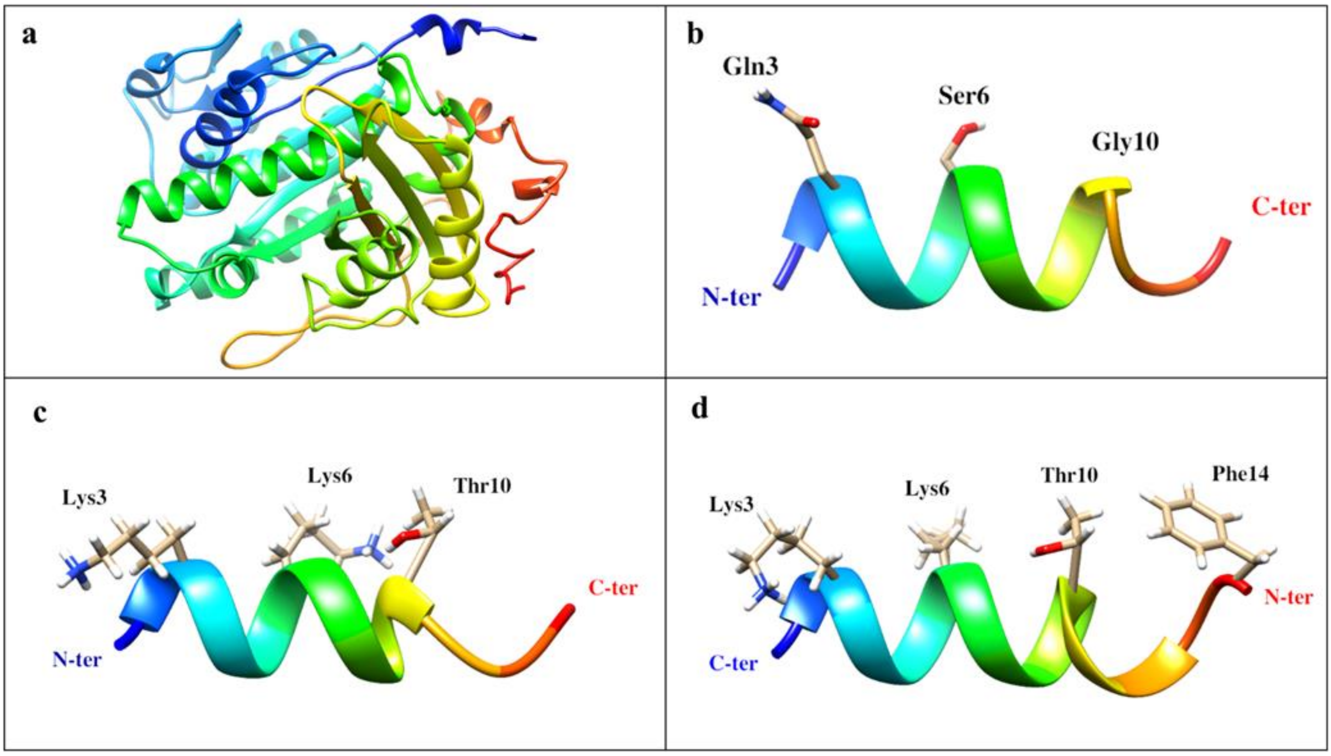

Figure S1. 3D model of FtsZ (**a**), native Temporin-L (**b**) TRIL analogue (**c**) and TRILF analogue (**d**) obtained by *ab initio* modelling with I-TASSER server.

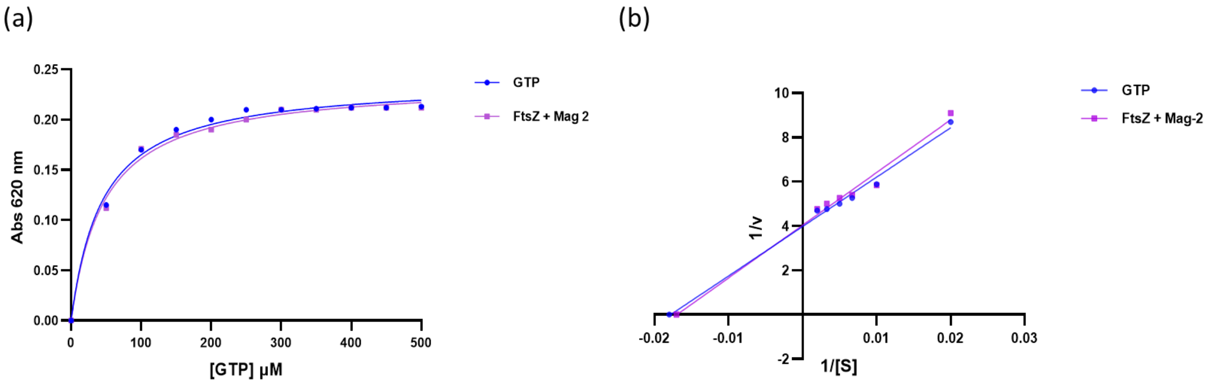

(c)

	GTP	Mag-2
Km	44,87 ± 4,91	47± 4.87
vmax	0,239 ± 0,005	0,238 ± 0,005

Figure S2. a) Enzymatic activity of recombinant FtsZ (12 µM) was performed in 25 mM PIPES/NaOH (pH 6.8), 20mM $MgCl_2$, in the absence (blue line) and in the presence of 35µM of Mag-2 peptide (magenta line), using GTP as substrate. The reaction was performed for 10 min and the Pi release was determined by measuring the absorbance at 620 nm following 25 min incubation. **b**) Lineweaver Burk plots from which the K_M constants were calculated. **c**) Calculated K_M constants and Vmax for the enzymatic assays. The experiment was performed in duplicate and presented as mean ± standard error**.**
